# Running Demands in Sub-Elite Male Rugby Players: Do You Train Like You Play?

**DOI:** 10.3390/sports14070302

**Published:** 2026-07-16

**Authors:** Francesco Chiarello, Corrado Lupo, Damiano Li Volsi, Luca Beratto, Domenico Cherubini, Paolo Riccardo Brustio, Alexandru Nicolae Ungureanu

**Affiliations:** 1 Facultad de Deporte, UCAM Universidad Católica de Murcia, 30107 Guadalupe, Spain; fchiarello@alu.ucam.edu (F.C.);; 2 NeuroMuscular Function|Research Group, School of Exercise and Sport Sciences, University of Turin, 10126 Turin, Italy; corrado.lupo@unito.it (C.L.); alexandru.ungureanu@unito.it (A.N.U.); 3 Department of Medical Sciences, University of Turin, 10126 Turin, Italy; 4 Department of Clinical and Biological Sciences, University of Turin, 10043 Turin, Italy; 5 Department of Life Sciences and Systems Biology, University of Turin, 10123 Turin, Italy

**Keywords:** GNSS technology, rugby union, time-motion KPIs, linear mixed models, training specificity

## Abstract

This study investigated whether weekly training sessions replicate the running demands of match play in sub-elite rugby union athletes, examining the principle of training specificity across positional roles. A total of 30 male players from a sub-elite rugby union team participated, contributing 306 training and match data points. External load variables were collected using 18 Hz GNSS devices and analyzed across ten time-motion KPIs, including distance per minute, maximal speed, distance distribution across speed zones, and acceleration–deceleration profiles. Linear mixed models assessed differences between training and match play within forwards, backs, and scrum-halves. Results revealed significant mismatches between training and match running demands, with positional roles exhibiting distinct profiles. Forwards performed substantially lower running volumes and intensities in training, particularly in moderate-speed zones. Backs showed similar total volumes but markedly reduced high-intensity actions, including maximal speed, high-speed running, and severe decelerations. Scrum-halves displayed the greatest alignment, with no major discrepancies across conditions. These findings indicate that game-based training alone does not sufficiently reproduce match play running intensity for any position. Integrating targeted closed-skill blocks and supplementary high-intensity conditioning within predominantly game-based frameworks may help balance representativeness, repetition, and physical overload, supporting more effective preparation for the running demands of competition.

## 1. Introduction

In recent years, the number of publications on performance analysis (PA) in rugby union has increased constantly, as has the use of a scientific and analytic approach to the game in its key components [1,2,3,4,5]. In particular, empirical PA studies have focused on various aspects of game, including tendencies [5], quantification of work rate [6], development of models for player activity in relation to positional roles [7,8], comparisons between winning and losing teams [9], and profiling of individual and team performance [10]. From a practical perspective, PA allows coaches to objectively assess their team’s performance while also analyzing opponents to identify strengths and weaknesses for strategic advantage, including information regarding what, who, when, and where these behaviors happen [11]. A comprehensive PA framework should include both individual and team actions, providing a clear and precise performance profile that is both accurate and practicable. In addition, PA focused on positional information by means of the global navigation satellite system (GNSS) to analyze external load during official matches and training sessions [11], even in relation to technical and tactical data, thus providing an integrated approach able to make performance more easily understandable [7,12,13,14,15].

Moreover, PA is also used to investigate the consistency of the external load across both training sessions and official matches while accounting for physical as well as technical and tactical factors. Previous research has highlighted the importance of monitoring the training load carried out during the week, as it represents a big percentage of the athletes’ total workload [16]. In fact, systematic training-load monitoring enables strength and conditioning coaches to design an off-field training program to simulate game and positional roles’ specific demands [17,18,19]. Training specificity is crucial to accurately replicate performance demands during official competitions. Ideally, training sessions should closely mirror the technical, tactical, and physical demands of competition to optimize player readiness and effectiveness. In particular, the running demands in rugby union vary based on positional roles, with backs covering a greater running volume (i.e., total distance), reaching higher maximum speeds and greater numbers of sprints, while predominantly covering total distance at low speed [20]. On the other hand, forwards tend to have reduced running demands compared to backs because of their involvement in set pieces (e.g., lineout, scrum) and contacts (e.g., rucking, mauling, carrier support) [20]. Moreover, running demands are closely linked to technical and tactical events. Specifically, impacts and accelerations are more associated with ball carriers, while impacts and decelerations are primarily related to tackling [14]. Additionally, total distance covered and high-speed running are positively correlated with the number of passes and ball possession [15]. Therefore, training drills should be designed to mirror the demands of official matches, ensuring that players develop the required physical conditioning based on the specific requirements of their positional roles.

However, previous research has demonstrated a discrepancy between training workloads and actual match demands [2]. In fact, players cover a significantly greater distance per minute during matches compared to training sessions, although no significant differences were found in very high intensity activities (>20 km/h) between matches and training sessions. Based on the positional role, first and third row players cover significantly more distance in games than in training sessions, as well as outside backs [2]. Additionally, variations in performance between matches and training sessions were influenced by both the duration of the interval between games and the result of the preceding match [21]. However, this research focuses on elite rugby players, and the phenomenon remains unexplored among sub-elite athletes. In fact, elite and sub-elite rugby might represent distinct competitive contexts, and findings from elite players may not fully reflect sub-elite demands [22].

Therefore, this study aimed to explore the “train like you play” principle to investigate the specificity of training session running demands compared with competitive match demands in sub-elite rugby union players. In particular, external load in official matches was compared with the training week load, according to the positional roles.

## 2. Materials and Methods

### 2.1. Participants

Thirty senior sub-elite players involved in the Italian Serie A1 Championship, including 15 forwards (age: 22 ± 3 years; height: 188.5 ± 7.9 cm; weight: 105.9 ± 10.9 kg), 11 backs (age: 23 ± 4 years; height: 175 ± 7.9 cm; weight: 85.3 ± 8.9 kg), and 4 scrum-halves (age: 20 ± 1 years; height: 172 ± 2.0 cm; weight: 78.0 ± 7.0 kg) participated in this study. All players were recruited from the same team and had at least 8 years of experience in rugby competitions and training, at the national or international level. Eighteen matches from the 2024–2025 Serie A season were included (wins: 7; draws: 2; loses: 9); written informed consent was obtained from recruited players, and the Club management approved the study. The local institutional review board approved this study (ID 0619881 29 October 2024).

### 2.2. Design and Procedures

Time-motion (Key Performance Indicators) KPI data were collected across the 2024–2025 season. All KPIs (Supplementary Table S1) were normalized and therefore independent of the number of training sessions completed by each player. For training, each KPI was first calculated for each individual training session. Session values were then averaged across all training sessions completed by each player during the week preceding the match. Because all KPIs were expressed as relative (e.g., m/min) or percentage-based variables, the resulting weekly value represented the player’s mean running demand during the training microcycle, independent of the number of training sessions completed. To ensure consistency within the database, only match (n = 153) and training data points (n = 153) from players who participated in official matches for at least 40 min were included in the analyses. Additionally, only players who participated in at least one full training session within the five days preceding the match were included. The number of matches played per athlete varied across the season, ranging from 1 to 10 matches per player, due to injuries, team rotation strategies, and failure to meet the minimum inclusion criteria for match and training exposure. Because of the different performance profiles [23,24,25], all analyses were conducted separately for forwards (match and training performances: n = 150), backs (match and training performances: n = 118), and scrum-halves (match and training performances: n = 38). Although scrum-halves are typically classified as backs, their data were analyzed independently due to their distinct performance profile, particularly their high specialization in passing and playmaking, which often makes them statistical outliers [25].

The external training load was assessed using GPS (global positioning system) units equipped with an 18 Hz satellite signal, 120 Hz triaxial gyroscope and accelerometer (Gpexe Pro 2, Exelio S.R.L., Udine, Italy). The GPS units were securely attached to the players’ torsos beneath their official competitive t-shirts, and activated ~45 min before match kick-off and before each training session. The following external load variables were collected from GPS data during each session. Distance/min (m/min) represented the average distance covered per minute, expressed in meters per minute. Max Speed (km/h) indicated the maximum speed reached by a player during the session, while Max Acc (m/s^2^) represented the maximum acceleration achieved. Run distance (%) expressed the percentage of the total distance covered while running. The distribution of distance across speed zones over the total distance was calculated as follows: Distance speed Z1 (0–7.2 km/h), Z2 (7.3–14.4 km/h), Z3 (14.5–21.6 km/h), and Z4 (21.7–28.8 km/h). In addition, acceleration and deceleration demands were described using Distance acc Z2 (%), representing the percentage of total distance covered while accelerating above 2.5 m/s^2^, and Distance dec Z2 (%), representing the percentage covered while decelerating below −2.5 m/s^2^ over the total distance.

### 2.3. Statistical Analysis

Means and standard deviations were calculated for each player group (forwards, backs, and scrum halves) across all KPIs (Table 1). Each time-motion KPI was normalized based on either total time in play or total distance, as recommended by Hughes & Bartlett [3]. Before aggregating individual training-session data (n = 1831) to summarize the week prior to the official match, outliers were identified and removed (n = 92; i.e., 5%) if they exceeded a three-standard deviation (z-score ≥ 3) threshold, calculated separately for each role (i.e., forwards, backs, and scrum halves) and event type (i.e., training session and official match). This procedure accounted for positional and event-specific running profiles while limiting the influence of extreme observations. It was specifically implemented to remove GNSS recording artefacts, as visual inspection showed that some excluded observations were clearly implausible.

To assess differences in time-motion KPIs between matches and training sessions, a series of Linear Mixed Models (LMMs) was performed. Specifically, the positional roles, training/match type, and their interactions were included as fixed effects, while the 10 time-motion KPIs served as dependent variables (see Formula (1) for an example of an LMM).

*Time-motion KPI Dependent Variable* ~ β_0_ + β_1_ (Type) + β_2_ (Role) + β_3_ (Role × Type) + (1|player) + ε (1)

Formula (1). Example of the linear mixed model utilized in this study.

When significant interaction effects were observed, Bonferroni-adjusted post hoc comparisons were performed to control the family-wise Type I error rate across multiple pairwise comparisons. Players were included as a random intercept to account for the repeated observations collected from each participant throughout the season. Model diagnostics were performed for each linear mixed model. Model convergence was verified, and residual distributions were assessed using both formal normality tests and visual inspection of residual histograms and Q–Q plots. Homoscedasticity was evaluated by visually inspecting residual-versus-fitted plots. Although minor deviations from normality were observed in the tails of the residual distributions for 2 (i.e., meters per min, max acceleration) of the 10 models, no substantial violations were identified that were considered likely to compromise model validity or interpretation. For linear mixed models, effect magnitude was evaluated using marginal and conditional R^2^ values, reflecting the variance explained by fixed effects alone and by the full model, respectively [26]. The level of significance was set at *p* = 0.05. Statistical analysis was conducted using the statistical software Jamovi Version 2.3.21.0.3.

## 3. Results

Table 1 reports descriptive statistics (mean ± SD) of all TMA KPIs across positional group (i.e., forwards, backs and scrum-halves).

**Figure 1:**
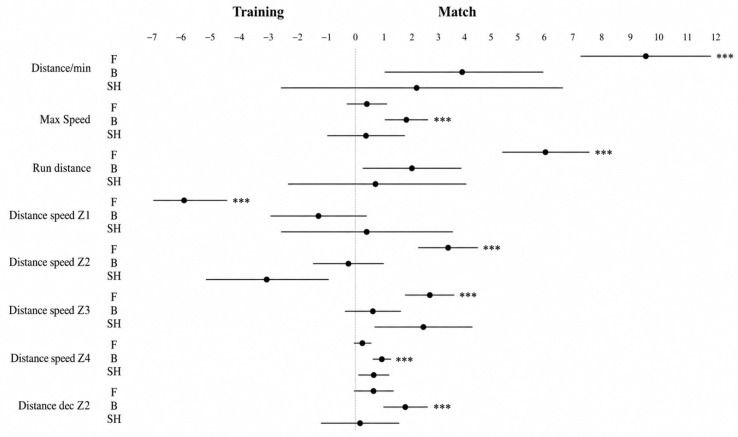
Post hoc pairwise contrasts (Training-Match) within each positional role for the significantly different time-motion KPIs, adjusted using the Bonferroni correction. F = forwards; B = backs; SH = scrum halves; *** *p* < 0.001.

Linear mixed model results of the time-motion KPIs revealed several significant *Role* × *Type* interactions. Specifically, significant interactions were found for Distance/min (F_(2, 272.0)_ = 7.27, *p* < 0.001, R^2^m = 0.43, R^2^c = 0.44), Max Speed (F_(2, 265.3)_ = 3.98, *p* = 0.020, R^2^m = 0.29, R^2^c = 0.63), Run distance (F_(2, 271.5)_ = 10.22, *p* < 0.001, R^2^m = 0.30, R^2^c = 0.48), and for distances in Z1 (F_(2, 271.1)_ = 11.36, *p* < 0.001, R^2^m = 0.27; R^2^c = 0.48), Z2 (F_(2, 271.2)_ = 16.89, *p* < 0.001, R^2^m = 0.11; R^2^c = 0.50), Z3 (F_(2, 271.8)_ = 4.50, *p* = 0.012, R^2^m = 0.48; R^2^c = 0.57), and Z4 (F_(2, 272.8)_ = 5.82, *p* = 0.003, R^2^m = 0.38; R^2^c = 0.67) speed zones, indicating that the effect of *Type* (i.e., training vs. match) differed across playing roles. No significant interactions were found for Max Acc (F_(2, 269.8)_ = 0.94, *p* = 0.393) or Distance acc Z2 (F_(2, 272.9)_ = 2.28, *p* = 0.104), while the interaction for Distance dec Z2 approached significance (F_(2, 271.3)_ = 2.97, *p* = 0.053). Post hoc pairwise comparisons for the main effects within these interactions are presented in Figure 1. Detailed statistical results are reported in Supplementary Table S2. Complete fixed-effect parameter estimates with their 95% confidence intervals are reported in Supplementary Table S3.

## 4. Discussion

This study explored the principle of specificity regarding running demands characterizing training session external load in comparison with competitive match demands in sub-elite rugby union players. In particular, external loads from pre-match training sessions were compared to those of actual matches across a full season and for all the positional roles. The main finding of this study is that external load varies between training and match play according to the positional roles. Overall, running demands during training are lower, disadvantaging forwards in terms of volume and density and backs according to high-intensity efforts. No significant differences were observed for scrum halves. However, this finding should be interpreted with caution, as the limited number of scrum-halves included in the sample (4 subjects and 38 data points) may have reduced the statistical power to detect meaningful differences between training and match play for this position. Forwards recorded higher distance per minute, running distance, and Z2 and Z3 distance, while backs consistently achieved higher maximal speeds and higher distance while decelerating below −2.5 m/s (Distance dec Z2) in match play. Although this phenomenon has previously been investigated in rugby [2,27,28], to our knowledge this is the first study that has investigated it in sub-elite players, with particular attention on profiling volume according to specific levels (distance covered at different speed and acceleration/deceleration levels). This approach provides a deeper understanding of how distance distribution across speed zones differs between positional roles.

In fact, training specificity is essential to replicate the technical, tactical, and physical demands of competition [29], and it should closely mirror the demands of competition to optimize players’ effectiveness. Furthermore, accounting for performance level, such as elite versus sub-elite athletes, is crucial, given the substantial differences in anthropometry, technical abilities, and injury likelihood between groups [30,31]. Nevertheless, training design should balance the replication of match demands with other important objectives, including periodization, recovery, injury prevention, and technical and tactical development. Therefore, the present findings should be interpreted as referring specifically to the running demands quantified through GNSS-derived variables. Moreover, from the external load perspective, running demands in rugby union vary according to the positional roles, with forwards combining running with almost static high intensity demands such as scrumming, tackling, rucking, and mauling [20,23]. Specifically, forwards tend to cover less absolute and relative distance (i.e., m/min) than the backs, as well as less peak speed and distance at high speed (Z4 and above) [32]. On the other hand, backs generally cover a greater running volume (i.e., total distance) and density (i.e., distance per minute), reaching higher maximum speeds, while predominantly covering total distance at low speed [32]. Finally, scrum halves cover the most total distance at the highest average speed [20,32].

Our results are consistent with the running profiles for all the positional roles in professional rugby union, suggesting that sub-elite players exhibit comparable patterns during matches. In fact, both elite and sub-elite players show the same running pattern: forwards complete lower running volumes during training, while backs reach lower maximal speeds [20,32]. Conversely, elite players, both forwards and backs, tend to perform a greater number of sprints during training compared with match play [2]. However, in this study notable discrepancies between training and match play were observed. The running demand profiles of forwards and backs were most affected. Milder training sessions appeared to constrain forwards’ activity profiles, leading to reduced time spent jogging and striding (i.e., zones Z2 and Z3) and increased time standing or walking. Because static exertions such as scrummaging, tackling, and lineouts were not accounted for in the analysis, lower running distances cannot be interpreted as rest periods. However, these static exertions may partially explain the lower running density (i.e., distance per minute). Nonetheless, these sessions did not fully reproduce the running demands observed during match play, where players are required to perform both static and dynamic high-intensity efforts.

Conversely, for backs, training sessions did not fully reproduce the high-speed running demands observed during match play, particularly with respect to distance covered in zone Z4 and maximal running speed. Furthermore, severe deceleration distances were consistently lower in training than in match play. Since such decelerations have been shown to be closely associated with key technical and tactical events such as tackling [14,15], this suggests that training sessions tend to be more conservative in terms of physical contact and collision intensity compared to actual match situations. Although this strategy helps minimize the negative effects of replicating match demands during training, it may also limit exposure to some of the high-speed running activities encountered during competition.

According to this study in sub-elite players, as well as others in elite players [2,27], different strategies could be implemented to achieve match play demands during training sessions. First, game-based training may be chosen because it allows coaches to target two objectives simultaneously: developing technical and tactical skills while exposing players to specific running demands [27]. Moreover, this approach may be even more beneficial for sub-elite players, whose tactical understanding and technical proficiency still need to be developed and who therefore have greater margin for improvement. In fact, game-based environments expose athletes to realistic perceptual–action couplings, requiring them to read the game, coordinate with teammates, and adapt their skills under variable constraints. This might accelerate tactical understanding and situational awareness, areas where sub-elite players often lag and thus have the greatest potential for improvement [33,34]. However, within this scenario, certain skills that require a high number of repetitions, such as scrummaging, may be undertrained. In fact, practical experience suggests that repeated practice is required to automate posture, strength, and stability during pushing or positional control for those skills. Although an 80 min match includes roughly 20 scrum events, averaging only one every four minutes, a closed-skill training session can achieve the same number of repetitions in approximately 20 min [25]. Consequently, a closed-skill session focused on scrummaging, lineouts, or set pieces may be more effective from a technical and tactical point of view.

Secondly, a more closed-skill-oriented training session can enhance technical proficiency and help automate strategic behaviors such as set pieces, lineouts, and scrummaging, thereby contributing to performance improvements. However, this approach may not allow players to meet match play running demands [2,27]. To address this, integrating targeted closed-skill blocks within a predominantly game-based approach, alongside additional high-intensity conditioning, can better balance representativeness, repetition, and physical load [27,33]. Conditioning work also enables coaching staff to compensate for the variable undertrained during closed-skill sessions, such as running volume for forwards or maximal-speed exposure for backs, as highlighted in this study.

From a practical perspective, the present findings highlight the importance of designing positional role-specific training strategies in sub-elite rugby union. Weekly training sessions did not consistently replicate the running demands of match play, suggesting that coaches should complement traditional practice with targeted interventions according to positional role. Starting from these results and according to the existing literature, forwards may benefit from additional running volume, whereas backs require greater exposure to maximal-speed running and high-intensity deceleration actions. A balanced combination of game-based activities, closed-skill practice, and supplementary conditioning may therefore provide a more effective approach to simultaneously develop technical and tactical skills and reproduce match play running demands. Finally, regular monitoring of external load by positional role may help practitioners identify underexposed physical demands and adjust training accordingly to improve competition readiness.

## 5. Conclusions

This study examined the alignment between weekly training loads and match play running demands in sub-elite rugby union players, revealing that training sessions did not fully reproduce the high-speed running demands typically observed during competitive match play. Accordingly, game-based training alone did not appear to replicate match-level running demands, particularly for high-speed actions. A more comprehensive training model integrating representative game-based tasks with targeted closed-skill blocks and high-intensity conditioning may therefore represent a useful strategy to address the running-demand discrepancies identified in the present study. Such an approach may help practitioners balance technical and tactical objectives with adequate repetition and running overload, while increasing exposure to underrepresented running demands (e.g., running volume for forwards and maximal-speed exposure for backs).

This study presents several limitations that should be considered when interpreting the findings. First, all data were collected from a single sub-elite team, which may limit the generalizability of results to other competitive levels, training environments, or tactical systems. The training methodology, squad composition, and coaching philosophy of this team may have uniquely influenced the observed physical profiles. Second, contextual match variables, such as opposition strength, score margin, game location, and tactical strategy, were not incorporated into the analysis, despite their known influence on running and collision demands [35]. Third, the GNSS devices used could not reliably quantify contact events (e.g., tackling, rucking, scrummaging), which represent a substantial component of rugby union performance and may directly influence running outputs [36]. In addition, effective rugby preparation also involves decision-making, attention, and perception–action coupling, all of which contribute to the overall complexity of rugby performance and may influence perceived exertion and subsequent physical performance [37,38]. Although these aspects were not assessed in the present study, they should be considered when interpreting the observed discrepancies between training and match running demands. Representative training tasks may therefore provide benefits that extend beyond running external load, particularly by promoting decision-making, perception–action coupling, and sport-specific skill execution. Consequently, the present findings should be interpreted within the context of running demands and not as a comprehensive evaluation of training specificity in rugby union. Fourth, the small sample size of scrum-halves included in the study (n = 4) likely limited the statistical power to detect meaningful differences, which may explain the absence of significant differences between training and match play for this position. As a result, discrepancies between training and match loads may partially reflect unmeasured collision demands rather than differences in running activity only. For these reasons, future studies should integrate multi-team samples, technical and tactical indicators, and contextual performance indicators in sub-elite environments.

## Figures and Tables

**Table 1:** Means and standard deviations of all time-motion KPIs within each player group (forwards, backs, and scrum halves) during training sessions and matches.

KPI	Forwards		Backs		Scrum-Halves	
	Training	Match	Training	Match	Training	Match
Distance/min (m/min)	52.8 ± 4.0	62.4 ± 9.0	65.5 ± 5.4	69.2 ± 9.4	68.6 ± 5.8	70.8 ± 9.7
Max Speed (km/h)	23.5 ± 1.8	23.9 ± 3.7	25.6 ± 1.7	27.3 ± 2.5	25.3 ± 2.0	25.6 ± 4.3
Max Acc (m/s^2^)	4.0 ± 0.3	4.1 ± 0.8	4.2 ± 0.3	4.4 ± 0.5	4.2 ± 0.3	4.1 ± 0.7
Run distance (%)	36.6 ± 5.1	43.1 ± 6.7	43.3 ± 2.6	45.2 ± 5.7	45.1 ± 4.1	45.9 ± 7.3
Distance speed Z1 (%)	61.0 ± 4.7	55.1 ± 6.7	54.4 ± 2.7	53.2 ± 6.1	52.4 ± 4.2	52.8 ± 7.2
Distance speed Z2 (%)	30.0 ± 3.4	33.2 ± 5.1	29.9 ± 3.3	29.7 ± 5.7	31.7 ± 4.0	28.7 ± 3.3
Distance speed Z3 (%)	8.2 ± 2.7	10.7 ± 3.3	13.6 ± 1.9	14.2 ± 2.4	13.9 ± 3.5	16.3 ± 4.4
Distance speed Z4 (%)	0.6 ± 0.5	0.9 ± 0.8	1.9 ± 1.2	2.8 ± 1.6	1.6 ± 1.3	2.2 ± 1.6
Distance acc Z2 (%)	0.8 ± 0.2	0.7 ± 0.2	1.2 ± 0.3	1.0 ± 0.3	0.9 ± 0.2	0.7 ± 0.2
Distance dec Z2 (%)	0.7 ± 0.2	0.8 ± 0.3	1.0 ± 0.3	1.2 ± 0.3	0.8 ± 0.3	0.8 ± 0.3

## Data Availability

The raw data supporting the conclusions of this article will be made available by the authors on request.

## Supplementary Materials

The following supporting information can be downloaded at https://www.mdpi.com/article/10.3390/sports14070302/s1, Table S1: Description of the 10 time–motion KPIs analyzed, including distance covered at different intensity zones (Z1–Z4) and metrics related to accelerations and decelerations during training sessions and matches; Table S2: Results of Bonferroni-adjusted post hoc pairwise comparisons for the main effects within the Type × Role interactions across all key performance indicators (KPIs); Table S3: LMM results: fixed-effect estimates, 95% confidence intervals (CI), and associated *p*-values. Type: MATCH vs. TR (training); Role: B = backs, F = forwards (reference category), SH = scrum-halves.
