# Supplementary material for: Running Demands in Sub-Elite Male Rugby Players: Do You Train Like You Play?
Source: Sports (Basel). 2026 Jul 16;14(7):302. doi: 10.3390/sports14070302 (PMC13416986; doi:10.3390/sports14070302)
Supplement: Supplementary file 1 [file sports-14-00302-s001.zip › sports-4388640-supplementary.pdf]

Table S1. Description of the 10 time–motion KPIs analyzed, including distance covered at different intensity zones (Z1–Z4) and metrics related to accelerations and decelerations during training sessions and matches.

Time-motion KPI	Description
Distance metres per min (m/min)	Average distance covered by a player per minute during a session (m/min).
Run distance (%)	Percentage of the total distance covered by a player during a session while running.
Max Speed (km/h)	Maximum speed reached by a player during a session, expressed in km/h.
Max Acc (m/s^2)	Maximum acceleration reached by a player during a session, expressed in m/s^2.
Distance speed Z1 (%)	Percentage of the total distance covered at speeds between 0 and 7.2 km/h.
Distance speed Z2 (%)	Percentage of the total distance covered at speeds between 7.2 and 14.4 km/h.
Distance speed Z3 (%)	Percentage of the total distance covered at speeds between 14.4 and 21.6 km/h.
Distance speed Z4 (%)	Percentage of the total distance covered at speeds between 21.6 and 28.8 km/h.
Distance acc Z2/total distance (%)	Percentage of the total distance covered while accelerating at values greater than 2.5 m/s^2.
Distance dec Z2/total distance (%)	Percentage of the total distance covered while decelerating at values lower than −2.5 m/s^2.

Table S2. Results of Bonferroni-adjusted post hoc pairwise comparisons for the main effects within the Type × Role interactions across all key performance indicators (KPIs).

KPI	Type*Role	Difference	SE	t	$P_{Bonferroni}$
Distance/min (m/min)	M F – TR F	9.658	1.19	8.092	<0.001
	M B – TR B	3.655	1.35	2.714	0.106
	M SH – TR SH	2.119	2.36	0.897	1.000
Max Speed (km/h)	M F – TR F	0.416	0.339	1.225	1.000
	M B – TR B	1.762	0.384	4.593	<0.001
	M SH – TR SH	0.371	0.676	0.549	1.000
Run distance (%)	M F – TR F	6.4681	0.762	8.4900	< .001
	M B – TR B	1.933	0.860	2.249	0.380
	M SH – TR SH	0.737	1.508	0.488	1.000
Distance speed Z1 (%)	M F – TR F	-5.847	0.756	-7.738	< .001
	M B – TR B	-1.250	0.853	-1.465	1.000
	M SH – TR SH	0.408	1.496	0.273	1.000
Distance speed Z2 (%)	M F – TR F	3.188	0.540	5.901	< .001
	M B – TR B	-0.206	0.610	-0.338	1.000
	M SH – TR SH	-2.993	1.069	-2.798	0.082
Distance speed Z3 (%)	M F – TR F	2.549	0.430	5.933	< .001
	M B – TR B	0.621	0.487	1.276	1.000
	M SH – TR SH	2.341	0.854	2.743	0.097
Distance speed Z4 (%)	M F – TR F	0.231	0.135	1.707	1.000
	M B – TR B	0.920	0.152	6.064	< .001
	M SH – TR SH	0.638	0.267	2.385	0.266
Distance dec Z2 (%)	M F – TR F	0.063	0.034	1.827	1.000
	M B – TR B	0.171	0.039	4.385	< .001
	M SH – TR SH	0.016	0.068	0.235	1.000

Table S3. LMM results: fixed-effect estimates, 95% confidence intervals (CI), and associated *p*-values. Type: MATCH vs. TR (training); Role: B = backs, F = forwards (reference category), SH = scrum-halves.

KPI	Fixed effect	Estimate	95% CI	p
Distance/min (m·min⁻¹)	MATCH − TR	5.13	3.18 to 7.07	<0.001
	B − F	9.76	7.80 to 11.73	<0.001
	SH − F	12.17	9.27 to 15.07	<0.001
	MATCH − TR × B − F	−5.95	−9.49 to −2.41	0.001
	MATCH − TR × SH − F	−7.49	−12.69 to −2.28	0.005
Max Speed (km·h⁻¹)	MATCH − TR	0.84	0.28 to 1.39	0.003
	B − F	3.53	1.90 to 5.16	<0.001
	SH − F	3.21	0.89 to 5.53	0.012
	MATCH − TR × B − F	1.38	0.37 to 2.38	0.008
	MATCH − TR × SH − F	−0.01	−1.50 to 1.47	0.987
Max Acc (m·s⁻²)	MATCH − TR	0.09	−0.04 to 0.22	0.172
	B − F	0.31	0.09 to 0.53	0.012
	SH − F	0.26	−0.07 to 0.58	0.130
	MATCH − TR × B − F	0.07	−0.16 to 0.30	0.565
	MATCH − TR × SH − F	−0.18	−0.51 to 0.16	0.312
Run distance (%)	MATCH − TR	3.03	1.79 to 4.27	<0.001
	B − F	4.78	2.24 to 7.32	0.001
	SH − F	6.14	2.50 to 9.78	0.003
	MATCH − TR × B − F	−4.49	−6.75 to −2.23	<0.001
	MATCH − TR × SH − F	−5.69	−9.00 to −2.37	<0.001
Distance speed Z1 (%)	MATCH − TR	−2.21	−3.44 to −0.98	<0.001
	B − F	−4.67	−7.27 to −2.07	0.002
	SH − F	−5.91	−9.63 to −2.18	0.004
	MATCH − TR × B − F	4.55	2.31 to 6.79	<0.001
	MATCH − TR × SH − F	6.21	2.92 to 9.50	<0.001
Distance speed Z2 (%)	MATCH − TR	−0.01	−0.89 to 0.87	0.986
	B − F	−1.75	−4.19 to 0.70	0.173
	SH − F	−1.93	−5.42 to 1.55	0.285
	MATCH − TR × B − F	−3.38	−4.99 to −1.78	<0.001
	MATCH − TR × SH − F	−6.17	−8.52 to −3.82	<0.001
Distance speed Z3 (%)	MATCH − TR	1.83	1.12 to 2.53	<0.001
	B − F	4.73	3.57 to 5.89	<0.001
	SH − F	5.98	4.30 to 7.66	<0.001
	MATCH − TR × B − F	−1.89	−3.17 to −0.62	0.004
	MATCH − TR × SH − F	−0.17	−2.05 to 1.70	0.857
Distance speed Z4 (%)	MATCH − TR	0.60	0.38 to 0.82	<0.001
	B − F	1.63	0.98 to 2.27	<0.001
	SH − F	1.63	0.71 to 2.54	0.002
	MATCH − TR × B − F	0.69	0.29 to 1.09	<0.001
	MATCH − TR × SH − F	0.41	−0.18 to 1.00	0.174

KPI	Fixed effect	Estimate	95% CI	p
Distance acc Z2 (%)	MATCH − TR	−0.133	−0.188 to −0.078	<0.001
	B − F	0.332	0.194 to 0.469	<0.001
	SH − F	0.113	−0.083 to 0.310	0.268
	MATCH − TR × B − F	−0.107	−0.207 to −0.007	0.037
	MATCH − TR × SH − F	−0.077	−0.223 to 0.070	0.306
Distance dec Z2 (%)	MATCH − TR	0.084	0.027 to 0.140	0.004
	B − F	0.348	0.196 to 0.500	<0.001
	SH − F	0.207	−0.010 to 0.424	0.072
	MATCH − TR × B − F	0.108	0.005 to 0.211	0.040
	MATCH − TR × SH − F	−0.048	−0.198 to 0.103	0.538
